# Predictors of neurodevelopment outcomes among children under 5 years old in Nairobi informal settlements

**DOI:** 10.3389/fnins.2025.1623064

**Published:** 2025-11-04

**Authors:** Patrick Amboka, Margaret Nampijja, Peninah Masibo

**Affiliations:** ^1^African Population and Health Research Center, APHRC, Nairobi, Kenya; ^2^Amref International University, Nairobi, Kiribati; ^3^University of California San Francisco, San Francisco, CA, United States

**Keywords:** neurodevelopmental assessment, under five children, poor neurodevelopmental outcome, delayed neurocognitive recovery, slums (informal settlements), cognitive development

## Abstract

**Background:**

A child’s early years of life are a critical period for brain development, and malnutrition at this stage is associated with irreversible developmental delays. Developmental delays can be affected by maternal, child, and household factors. This study examined the association between acute malnutrition relapse and neurodevelopmental outcomes among children under 5 years old in Nairobi’s informal settlements.

**Methodology:**

The study used a comparative cross-sectional design that was hospital-based and was carried out in Viwandani and Korogocho informal settlements in Nairobi, Kenya. The three groups that were compared were children under 5 years old who had never suffered acute malnutrition, those who had the first episode of acute malnutrition, and those who had acute malnutrition relapse. Relapse in acute malnutrition was defined as wasting within 6 months after exiting treatment, as per the recommended discharge criteria (Council of Research and Technical Advice for Acute Malnutrition, CORTASAM). The study used both quantitative and qualitative methods, and the purposive sampling technique was used in both methods. The Malawi Development Assessment Tool (MDAT) assessed the neurodevelopment outcomes. Quantitative data is presented at the univariate, bivariate, and multivariate levels. Qualitative data were analyzed thematically, and predetermined themes and emerging themes were identified and added.

**Results:**

Acute malnutrition relapse was a significant predictor of neurodevelopment outcomes among children under 5 years old. Children who had either never relapsed from acute malnutrition or had normal nutrition status were 2.08 times more likely to have normal neurodevelopmental outcomes compared to children who had relapsed [AOR = 2.08; CI:1.11, 3.90; *p*-value, 0.02]. Maternal postpartum depression and child maturity status at birth were also significant predictors of neurodevelopmental outcomes among children under 5 years in Nairobi informal settlements [AOR = 3.62; CI:1.86, 7.04; *p*-value < 0.001], [AOR = 2.93; CI:1.21, 7.12; *p*-value, 0.02], respectively. The qualitative findings further showed that breastfeeding, sanitation, and diarrhea have an association with children’s neurodevelopmental outcomes.

**Conclusion:**

This study demonstrates that children under five who experience relapses due to acute malnutrition exhibit significantly delayed neurodevelopmental outcomes compared to those who do not relapse. Furthermore, qualitative findings showed that breastfeeding, sanitation, and diarrhea have an association with children’s neurodevelopmental outcomes.

## Introduction

For children to reach their full potential, they need five indivisible and interrelated components of nurturing care: adequate nutrition, good health, responsive caregiving, safety and security, and opportunities for learning (UNICEF, 2022). Adequate nutrition and good health are key to children’s physical and neurodevelopment (UNICEF, 2022). However, nutrition deficiencies remain among the top 20 causes of diseases in Kenya (the global burden of disease, GBD) measured in Disability-Adjusted Life Years (DALYs) (Debaprasad and Nira, 2025).

Acute malnutrition is a form of undernutrition that results from decreased intake of protein and/or energy and/or loss due to illness, resulting in bilateral pitting oedema and/or sudden weight loss. It is defined by the presence of bilateral pitting oedema or wasting [low Mid Upper Arm Circumference (MUAC) or low Weight-For-Height (WFH), Weight-For-Length (WFL)]. Moderate acute malnutrition (MAM) is defined by a weight for height/length (WHZ) indicator between −2 and −3 z-scores (standard deviations, SD) of the WHO international standard cut-off, or by a mid-upper arm circumference (MUAC) between 12.5 cm and 11.5 cm, or both (Fiorentino et al., 2016). Severe acute malnutrition (SAM) is a type of acute malnutrition where the MUAC < 11.5 CM or WHZ < −3 SD or the presence of bilateral pitting oedema (nutritional oedema), or both (Fiorentino et al., 2016). Acute malnutrition relapses refer to wasting within 6 months after exiting treatment, as per the recommended discharge criteria (Council of Research and Technical Advice for Acute Malnutrition, CORTASAN).

Globally, malnutrition is a cause of more than 3 million under-5 deaths annually (Kassaw et al., 2021). Sub-Saharan Africa (SSA) has a large percentage of children suffering from acute malnutrition, 13 million (Munthali et al., 2015). Thirty-five thousand deaths that occur among children under 5 years in Kenya are due to malnutrition (Fanzo et al., 2022). Between 2018 and 2019, the prevalence of acute malnutrition in Nairobi’s informal settlements was at a medium threshold but approaching high (9.93%) among children under 5 (De Vita et al., 2019). The United Nations clearly states that unless the world tackles inequality and poverty, 3 billion people worldwide will be living in slums by 2050 (Bhalla, 2023). In SSA, 62% of the population in urban areas dwell in slums (UN-Habitat, 2014). Nairobi city has around 40 slum areas, and around 56% of the city population lives in the slums (Mberu et al., 2016). These settlements have inadequate clean water, inadequate health services, and poor sanitation, creating an environment where children under 5 are highly susceptible to malnutrition because of the limited access to nutritious foods and suboptimal living conditions (Kimani-Murage et al., 2014).

Neurodevelopmental outcomes comprise fine motor skills, gross motor skills, cognitive and social skills, and language. Fine motor skills are the movement and function of the small muscles of the hands, fingers, tongue, lips, toes, and wrists. Gross motor skills refer to the abilities of large muscle control (for crawling, sitting, running, walking, and head movements). Social skills are abilities the children require to interact with others and understand themselves; they include showing frustrations, responsive smiling, recognizing themselves in the mirror, and preferring their primary caregiver to others (Komutambo et al., 2022). Language includes abilities in receptive language, expressive language, and articulation. Infants experience a significant improvement in motor development by 3–4 months of age (Hadders-Algra, 2018). Children between 2 and 6 months of age had higher chances of achieving the highest level of neurodevelopmental outcomes compared to children aged 19–23 months (Komutambo et al., 2022).

Studies that have been done across the world show that poor neurodevelopmental outcomes are indeed a problem. Statistics on the rate of poor neurodevelopmental outcomes among preterm babies in a study done in France showed that the rate was 28% (Pierrat et al., 2021). In Eastern Uganda, results showed that infants who had poor neurodevelopmental outcomes in at least one domain were 12.7% (Namazzi et al., 2019). Studies among children with normal weight for length/height showed that Kenya had a 4.47% prevalence of poor neurodevelopmental outcomes in 2022 (Abuga et al., 2022).

A child’s early years of life (0–5) are a critical period for brain development. A study done in the Philippines, Brazil, and Jamaica showed that malnutrition during this time is associated with irreversible developmental delays (Grantham-McGregor et al., 2007). Previous community-based studies done in African countries, including South Africa and Tanzania, showed that malnutrition had a significant association with poor neurodevelopmental outcomes in children under 5 (McCormick et al., 2020). In Uganda and Malawi, children under 5 years old who never recovered from wasting had poor neurodevelopmental outcomes (Babikako et al., 2022). In western Kenya, malnutrition was reported to be a significant predictor of delayed developmental milestones with an adjusted odds ratio of 13.9 (Gudu et al., 2020).

The various maternal factors that affect a child’s neurodevelopmental outcomes among children under 5 years include maternal education level, maternal age, maternal nutrition status, and maternal substance use during pregnancy/breastfeeding, and the contributors to maternal health (antenatal care (ANC) attendance, delivery care practices, and postnatal practices). The maternal factors are critical because brain development starts during the third week of gestation (Konkel, 2018). A study in Iraq found that there was a relationship between the level of education of the mother and the child’s neurodevelopment (Alkhazrajy and Aldeen, 2017). Additionally, maternal nutrition status and substance use during pregnancy routinely compound the effects of cognitive neurodevelopment (Hamadani et al., 2014).

Child factors that affect neurodevelopmental outcomes include the age of the child, gender of the Child, diet quality, birthweight, child maturity status at birth (either premature or mature), child nutrition status, breastfeeding guidelines, and postnatal complications.

The influence of the child’s gender on poor neurodevelopment appears to be contradictory in the existing literature; Girls have better neurodevelopmental outcomes compared to boys with the same nutrition status (Moore, 2024). Diet quality is positively associated with good neurodevelopmental scores in children (Sato et al., 2022). For example, fortification of food with iron, zinc, calcium, vitamin A and B2, and proteins has been linked to improved cognitive outcomes (Prado et al., 2019). Lack of dietary protein can inhibit/delay neurodevelopment (Sato et al., 2022). In this regard, recent evidence suggests that animal protein supplementation is an essential part of children’s diets to prevent malnutrition and improve cognitive abilities (Pimpin et al., 2019).

By the age of 1 year, low birth weight (LBW) babies often experience delayed neurodevelopment (Sato et al., 2022). Although the relationship between LBW and neurodevelopment at 6 and 24 months of age is clearly demonstrated, evidence of the long-term effects on neurodevelopment is not consistent (Prado et al., 2019). Preterm infants are vulnerable to the effects of malnutrition, which severely impairs their later neurodevelopment, and improving their early protein-energy deficit can promote brain growth (McCormick et al., 2020).

Stunted children typically have poor immune systems, brain functioning, and development of organs, which delays various areas of neurodevelopment (Falster et al., 2018; Komutambo et al., 2022). Breastfeeding positively impacts the nutritional content of breast milk and maternal bonding (Krol and Grossmann, 2018). There’s a strong association between lengths of exclusive breastfeeding and improved developmental scores across all age groups and geographic locations (Onyango et al., 2022). Child post-neonatal complications, such as inability to feed well, unconsciousness, hypothermia, convulsions, high body temperatures, and intra-uterine growth restriction, are associated with poor neurodevelopmental outcomes (El Rafei et al., 2021).

Various household factors affect the child’s neurodevelopmental outcomes. They include family composition, household food security, socio-economic status, Water, Sanitation, and Hygiene (WASH) indicators at the household level, household parental practices and decision-making at the household level, and intimate partner violence within the household. Children from households having food security tend to have better neurodevelopmental outcomes than those from households that are food insecure (Komutambo et al., 2022). Neurodevelopmental progress in children aged 6–23 months could be partially explained by improved household food security (Frongillo et al., 2017; Gaul and Issartel, 2016). Household-level food security is also associated specifically with the achievement of motor developmental milestones (Geletu et al., 2019; Komutambo et al., 2022). Low socioeconomic status has an association with poor neurodevelopmental outcomes among undernourished children (Miller et al., 2015; Sudfeld et al., 2015; Yousafzai et al., 2014). The Home Observation for Measurement of the Environment (HOME) parameter is an indicator of the quality of the home environment (both material and social). There is a strong role of the home environment in the child’s neurodevelopmental outcomes, over and beyond socioeconomic factors and health factors (Nampijja et al., 2018).

The early developmental stages of children are crucial for their adult well-being. Parental practices at the household level and decision-making at the household level are important predictors of a child’s cognitive abilities (Malhi et al., 2018). This includes parental involvement in children starting solid feeding at 7–8 months of age, and therefore, they tend to have more interaction with caregivers during feeding time (Malhi et al., 2018). Children’s exposure to domestic violence at the household level is associated with long-lasting effects on the child’s neurodevelopment because their stress-related system and brain are usually susceptible to environmental stimuli (Mueller and Tronick, 2019).

There is insufficient data on the prevalence of neurodevelopmental outcomes among children under 5 years old in Kenya (Ministry of Health, 2015). Investigating the relationship between relapse incidents and neurodevelopmental outcomes is important as it directly addresses a critical public health concern that impacts a vulnerable population. There is research evidence on the impact of malnutrition on neurodevelopment, but a gap exists in understanding how relapse incidents might influence these (McCormick et al., 2020). This study contributes to filling the evidence gap, particularly regarding whether relapses result in a more adverse neurodevelopmental trajectory compared to a single episode of malnutrition. Studies have been done on the association between malnutrition and neurodevelopmental outcomes globally, in Africa, and in Kenya, but there has been less focus on informal settlements, a unique and vulnerable setting that deserves specific scrutiny. This study recognizes the need to not only address immediate physical health concerns but also the potential long-term consequences for neurocognitive development. It aimed to investigate the association between recurrences of post-treatment acute malnutrition and neurodevelopmental outcomes in children under 5 years old living in Nairobi’s informal settlements. This research addresses a critical knowledge gap in the association between relapse and neurodevelopmental outcomes and focuses on the potential long-term consequences of relapse, which are often under-researched. By examining the link between nutritional relapses and neurodevelopment, the study is based on the hypothesis that children who relapse from acute malnutrition have poorer neurodevelopmental outcomes compared to those experiencing their first episode, as well as the normal children. The study aimed to provide insights that can inform interventions aimed at sustained recovery and full child development in resource-constrained settings.

## Methodology

### Research design

This study used a comparative (analytical) cross-sectional design that was hospital-based (Wang and Cheng, 2020). The exposure and the outcome were assessed simultaneously. A comparative cross-sectional study was chosen because of the need to measure the association and compare the differences between the groups. We used both qualitative and quantitative approaches in data collection.

### Study setting

The study was conducted in four hospitals within Nairobi informal settlements (Viwandani -Mareba Hospital and Kwa Reuben Health Center; Korogocho- Korogocho Health Center and Ngomongo Hospital). These hospitals have nutrition clinics for managing acute malnutrition. The four hospitals were selected because they are located within the slums and serve residents of the slum areas. The rates of acute malnutrition in Korogocho and Viwandani slums are higher compared to an informal settlement like Kibera, which had 2.5% wasting rates (Kimani-Murage et al., 2015). These slum areas are among the areas that have the highest rates of acute malnutrition in Nairobi, and the high rates of malnutrition can be attributed to inaccessibility to clean water, high levels of food insecurity, poor sanitation, and healthcare services, creating an environment where children under 5 years have high chances of becoming malnourished (Kimani-Murage et al., 2014).

### Target population

The study included three comparative groups from similar socioeconomic backgrounds. The study’s target population was children under 5 years old living in the two informal settlements who had never suffered acute malnutrition, those who had acute malnutrition for the first time, and those who had acute malnutrition relapse after treatment. Acute malnutrition relapses in our context referred to wasting within 6 months after exiting treatment, as per the recommended discharge criteria (Council of Research and Technical Advice for Acute Malnutrition, CORTASAM). The time frame was ascertained by the trained research assistants through the hospital records and/or the child’s malnutrition clinic card. Wasting included severe and moderate wasting. Severe wasting is diagnosed by having a WHZ < −3 or MUAC < 115 mm, whereas moderate wasting is diagnosed by WHZ < −2 but > −3 or MUAC between 115 and < 125 mm (World Health Organization, 2013).

The discharge criteria used for exit in SAM were WHZ ≥ −2 or MUAC ≥ 125 mm and no oedema for at least 2 weeks, while the discharge criteria for MAM were WHZ ˃ –2 or MUAC ˃ 125 mm (World Health Organization, 2013).

Children who had a first episode of acute malnutrition, and those who had relapsed, were sampled at the nutrition clinics within the outpatient department, while those who had never suffered from acute malnutrition were sampled at the Maternal Child Health (MCH) clinics within the outpatient department.

### Inclusion criteria

1. Children under 5 years old who had been living in Korogocho and Viwandani within the past year.
2. Acute malnourished children under 5 years old who had their first episode of acute malnutrition.
3. Children under 5 years old who had acute malnutrition relapse.
4. Children under 5 years old had never suffered from acute malnutrition.

### Exclusion criteria

1. Children under 5 years old had conditions such as cerebral palsy, genetic syndrome, congenital heart disease, malabsorption cerebral palsy, etc.
2. Clinically unstable children under 5 years old during the assessment test.
3. Children under 5 years old who resided outside Korogocho and Viwandani.
4. Acute malnourished children under 5 years old who had never received treatment.

### Sample size determination

Fischer’s formula for comparative cross-sectional studies was used to determine the sample size (Charan and Biswas, 2013). The sample size was calculated based on the assumptions from a study done in Kenya, which found that the citation-given prevalence for any neurodevelopmental impairment was 12.7% (Experimental group) and 1.00% (Comparison group) (Dwivedi et al., 2018). Most studies reported the overall aggregated prevalence of neurodevelopmental impairment as a single outcome and not as separate domains.

Sample size, N per group = 89.

The total sample size for the three groups = 89 × 3 = 267.

### Sampling technique

Quantitative interviews with caregivers- A purposive sampling technique was employed where participants were selected based on their availability and eligibility to either group. This sampling method involved recruiting participants who met the inclusion criteria as they presented themselves until the sample size was attained. However, participation in the study could have been influenced by factors such as parental education or health-seeking behaviors, their availability, etc., which may have potentially led to a biased sample. Qualitative interviews with caregivers, healthcare providers, community health volunteers, and community health assistants- Quota sampling was used, and sampling stopped once the required number of subjects was attained.

### Data collection instruments

For the quantitative component, both structured and unstructured questionnaires were used for data collection. The questionnaire was uploaded to the Survey CTO mobile data collection platform to ensure accuracy, validity, and completeness.

Qualitative data collection was done using a focus group discussion guide (FGD), an in-depth interview (IDI) guide, and a key informant interview (KII) guide. Quantitative interviews were done using a structured and semi-structured questionnaire. The questionnaire and the guides were developed in English and translated into Swahili as well. Validation prior to data collection was done through pre-testing in another informal settlement with similar settings to check for clarity and consistency. The tool underwent further modifications based on feedback from the pilot.

Data was collected by five research assistants for a period of 3 months, and they underwent a one-week training prior to data collection. Qualitative data were programmed in the Survey CTO mobile data collection platform, and relevant quality checks were included. For quantitative data, the quality checks that were put in place include constraints and data validation prompts to minimize invalid or incomplete entries. For quality control, the data were reviewed daily after collection.

The data on neurodevelopmental outcomes were collected quantitatively and qualitatively. Quantitative data were collected using structured and semi-structured questions in the Malawi Developmental Assessment Tool (MDAT). The assessment involved observation with a checklist, getting a parental report, and directly assessing the child. The assessment was done by four experienced research assistants with previous experience in children’s neurodevelopmental outcomes assessment. They were trained for 1 week on assessing the children’s neurodevelopmental outcomes for standardization purposes. The research assistants had previous training in the Early Childhood Development (ECD). This tool is relevant in assessing the neurodevelopment milestones of children between 0 and 6 years in Malawi and elsewhere in Africa and was refined and robustly tested using detailed psychometric techniques (Gladstone et al., 2010). The tool was used in the English version because it was administered by qualified and experienced research assistants who understood English very well.

The tool has been well-validated to assess children’s neurodevelopmental milestones in African rural settings (Namazzi et al., 2019). The tool examines four areas of development: fine and gross motor skills, social skills, and language. The cognitive skills assessments are embedded in the areas of the child’s motor skills and language. All items within each domain were reported as either “pass” or “fail.” If the child fails to cooperate, it is reported as “unknown.” The tool has a sensitivity and a validity of 97% in predicting neurodevelopmental milestones of children from 0 to 6 years. On the child’s neurodevelopmental milestones, each domain had a cut-off score, and the child was considered to meet the cut-off points if their developmental score was above the cut-off (Komutambo et al., 2022).

### Validity testing

To ensure that the tools are used for neurodevelopmental assessment, nutritional assessment, and data collection are aligned with the study objectives, they were reviewed to confirm their relevance. The MDAT is considered locally appropriate and has been standardized on African populations. Therefore, it is suitable for assessing the participants. It has been well-validated to assess children’s neurodevelopmental milestones in African rural settings (Namazzi et al., 2019).

### Reliability testing

The neurodevelopmental assessment tools were administered to a subgroup of participants on two separate occasions to check for consistency in results. A high correlation between the scores was an indicator of good reliability. Since multiple assessors were involved, the researcher ensured that there was agreement in scoring and interpretation. Reliability checks were conducted by having different assessors independently rate the same children and then calculate inter-rater agreement.

The process of taking measurements such as MUAC, weight, and length/height was standardized to minimize variability. The tools and methods were piloted in Kibera South Health Center to avoid contamination of the study sample. The researcher implemented data quality checks during data collection and entry to identify and correct errors promptly. There was pilot testing of all tools with a small sample to identify any inconsistencies or ambiguities. The tools were modified based on feedback to enhance reliability.

### Recruitment of the study participants

The caregivers were approached at Mareba Health Centre (Viwandani), and Korogocho Health Center (Korogocho). Once the potential participants were identified, eligibility criteria were used to identify those who would participate in the study. Consents were sought from the study participants before administering the questionnaire.

### Data collection process

Data collection began after approval from the ethics committee and the hospitals’ administrations. This was done as planned to ensure accuracy and reliability. Initially, a sampling framework was established to identify participants representative of the target population, and data collection instruments, such as questionnaires and interview guides, were developed and piloted to refine clarity and usability. Trained research assistants then administered the tools following standardized protocols, with quality checks and supervisory oversight in place to address any discrepancies promptly. Data on the child’s height or length, weight, and MUAC measurements to determine nutritional status. Height/length was measured using an infantometer, while weight was measured using a weighing scale/salter scale, with the children having minimal clothing. The height/length was taken when the child was barefoot. The weighing scales were calibrated every day before commencement of the data collection. Both the weight and height were taken in duplicates, and their average was recorded. The weight and height/length were then changed to the z scores (WHZ) using the WHO WHZ reference chart to classify the severity of the malnutrition. Child MUAC was measured using infant MUAC tapes, and measurements were taken halfway between the acromion and olecranon process at the midpoint. The tape was checked so that it was not too tight or too loose. The recordings were rounded to one decimal place. Oedema was assessed using the thumbs on the child’s legs, feet, and face. The presence of oedema on the feet only was reported as grade 1 (+), both feet and legs were reported as grade 2 (++), and both feet, legs, hands, and face were reported as grade 3 (+++) (Gasparis et al., 2020). For non-pregnant women, weight and height were taken using a weighing scale and a stadiometer, respectively.

Maternal postpartum depression was assessed using the Edinburgh Postpartum Depression Scale (EPDS). This tool has been culturally adopted for use in Kenya. The tool was administered with qualified and experienced research assistants who had prior training on assessing postpartum depression. The participants’ responses to the 10 questions were scored based on the seriousness of the symptoms, while others were reverse-scored. The total score was found by adding together the scores for each of the 10 items. The respondents who scored above 13 were likely to be suffering from depression. This was the Kenya validation threshold for informal settlements that was adopted (Madeghe et al., 2016). The tool was translated into Swahili and administered in the Swahili language.

### Data analysis and presentation

#### Quantitative data

Data were analyzed using STATA version 17.0. Data cleaning was done using frequency distribution. Analysis was done at both the bivariate and multivariate levels. Descriptive statistics were conducted on the continuous/numeric and categorical variables, and data were described using standard deviations and means. Crude (unadjusted) correlations were examined between relapse and potential risk factors. Multivariable adjusted logistic regression analysis since the outcome (relapse or not, either pass or fail of the neurodevelopment assessment) is a binary outcome. The regression was employed to identify factors associated with a child’s neurodevelopmental outcomes.

Crude (unadjusted) correlations between neurodevelopmental scores (MDAT) and relapse and other potential predictors of neurodevelopmental outcome. Multivariate logistic regression was conducted to determine the association between acute malnutrition relapse after treatment and neurodevelopmental outcomes by adjusting for other factors. Anthropometric data were analyzed using the WHO Anthro software. Children with a WHZ z score of < −2SD and a MUAC of <12.5 cm were classified as having acute malnutrition (Hai et al., 2020). The mothers (not pregnant) with a body mass index (BMI) < 18.5 kg/m2 will be classified as acute malnutrition, while pregnant women were classified as having acute malnutrition with a MUAC of less than 23 cm (Kpewou et al., 2020).

The specific domains of neurodevelopment outcomes were assessed separately, with cognitive capacity outcomes being embedded in the fine motor skills and language assessments as per the MDAT tool. Using the tool, children were then scored in two ways. First, by generating a categorical score of either pass or fail. In case he/she fail two or more items in any domain at an age where at least 90% of the population of reference is expected to pass, then they were reported as failed.

The strength of association variables was calculated using the odds ratio (OR) with a 95% Confidence Interval (CI). The results presentation was done in tables, graphs, and narratives to facilitate interpretation and comparison.

#### Qualitative data

Qualitative data were analyzed thematically using NVivo software, using pre-determined themes as well as identifying and adding any emerging themes. The coding was done by two research assistants. To check for inter-reliability, the two research assistants coded a single transcript, and a coding comparison query was done using the Kappa coefficient. The coding comparison query showed that a Kappa coefficient was one and a coding agreement of 95%, which indicated consistency in the coding. Emerging concepts from the qualitative data were also analyzed using Leximancer software (Software using a large language model (LLM) to get concepts and interactions from the qualitative data).

#### Ethical consideration

The study strictly followed the ethical guidelines for human subject protection. It involved obtaining informed consent from all participants after the purpose, confidentiality, benefits, risks, and degree of involvement were explained to them in a language they clearly understood. The respondents thereafter signed the consent form to indicate that they agreed to be interviewed. For confidentiality, interviews were done in a private place, and only the project team was able to access the data. Data was anonymized before analysis. The respondents also agreed to an assessment to be done on the child. Approval from the AMREF University School of Graduate Studies, AMREF Ethics and Ethics and Science Research Committee (ESRC), the National Commission for Science, Technology, and Innovation (NACOSTI), and relevant authorities (Including health facility approvals) was sought before starting data collection. Approval reference number: AMREF-ESRC P1609/2024.

## Results

### Participants characteristics

There were 267 children under 5 years of age included in the study. The proportion of the participants that were male was 47.19% (*n* = 126), whereas the proportion of female children was 52.81% (*n* = 141). The proportion of children aged 2 years and below was 54.68 (*n* = 146) and those who were above 2 years were 45.32% (*n* = 121). The proportion of children under 5 years from Viwandani and Korogocho was 50.18% (*n* = 134) and 49.82% (*n* = 133) respectively. The participants were categorized into three groups: either never suffered from acute malnutrition, had their first episode of acute malnutrition, and had acute malnutrition relapse after treatment. Each group comprised 89 children under 5 years of age as shown in Table 1.

**Table 1:** Participants characteristics.

Characteristic	Category	Frequency (*n*)	Percentage (%)
Sex	Male	126	47.19%
	Female	141	52.81%
Total		267	100%
Age group	≤2 years	146	54.68%
	2–5 years	121	45.32%
Total		267	100%
Location	Viwandani	134	50.18%
	Korohocho	133	49.82%
Total		267	100%

### Neurodevelopmental outcomes among children

The neurodevelopment focused on four areas: fine and gross motor skills, social skills, and language. The cognitive skills assessments are embedded in the areas of the child’s motor skills and language. All items within each domain were reported as either “pass” or “fail.” If the child fails to cooperate, it is reported as “unknown.” On the child’s neurodevelopmental milestones, each domain had a cut-off score, and the child was considered to meet the cut-off points if their developmental score was above the cut-off. In case he/she fail two or more items in any domain at an age where at least 90% of the population of reference is expected to pass, then they were reported as failed.

The proportion of children who had poor neurodevelopment was 44.57% (*n* = 119), while the proportion of those with normal neurodevelopment was 55.43% (*n* = 148). The proportion of children under 5 years who had never suffered from acute malnutrition and had poor neurodevelopment was 37.08% (*n* = 33). The proportion of children who had the first episode of acute malnutrition and had poor neurodevelopment was 42.70% (*n* = 38). The proportion of children with acute malnutrition who relapsed after treatment and had poor neurodevelopment was 53.93% (*n* = 48), as shown in Table 2.

**Table 2:** Neurodevelopment outcomes among children.

	Normal neurodevelopment *n* (%)	Poor neurodevelopment *n* (%)	Total *n* (%)
Children under 5 years who had never suffered from acute malnutrition	56 (62.92)	33 (37.08)	89 (100)
Children under 5 years who had their first episode of acute malnutrition	51 (57.30)	38 (42.70)	89 (100)
Children under 5 years who had acute malnutrition relapse after treatment	42 (46.07)	48 (53.93)	89 (100)
Total	148 (55.43)	119 (44.57)	267 (100)

### Factors associated with neurodevelopmental outcomes among children

#### Socio-demographic factors

The proportion of primary caregivers with low education and children with poor neurodevelopmental outcomes was 23.53% (*n* = 28). The proportion of primary caregivers with low education levels and children with normal neurodevelopmental outcomes was 15.54% (*n* = 23). The proportion of children less than 2 years old who had poor neurodevelopmental outcomes was 60.50% (*n* = 72). The proportion of children less than 2 years old but had children with normal neurodevelopmental outcomes was 50.00% (*n* = 74). The proportion of female children with poor neurodevelopmental outcomes was 49.57% (*n* = 59), whereas the proportion who had normal neurodevelopmental outcomes was 55.41% (*n* = 82). There was no association between maternal education level, maternal age, child’s age, and gender and neurodevelopmental outcomes [COR = 1.38; CI:0.78, 2.45; *p*-value: 0.27], [COR = 1.01; CI:0.59, 1.72; *p*-value: 0.97],[COR = 1.53; CI:0.94, 2.50; *p*-value: 0.09], and [COR = 0.79; CI:0.49, 1.28; *p*-value: 0.34], respectively as shown in Table 3.

**Table 3:** Associations between socio-demographic factors and neurodevelopmental outcomes among children.

	Normal neurodevelopment *n* (%)	Poor neurodevelopment *n* (%)	COR	95% CI	*P*-value
Education level					
High education	125 (84.46)	91 (76.47)	1.38	0.78, 2.47	0.27
Low education	23 (15.54)	28 (23.53)			
Total	148 (100)	119 (100)			
Maternal age					
25 years and above	93 (62.84)	69 (57.98)	1.01	0.59, 1.72	0.97
Less than 25 years	55 (37.16)	50 (42.02)			
Total	148 (100)	119 (100)			
Child’s age					
2 years and above	74 (50.00)	47 (39.50)	1.53	0.94, 2.50	0.09
Less than 2 years	74 (50.00)	72 (60.50)			
Total	148 (100)	119 (100)			
Gender					
Male	66 (44.59)	60 (50.42)	0.79	0.49, 1.28	0.34
Female	82 (55.41)	59 (49.57)			
Total	148 (100)	119 (100)			

COR, Crude Odds Ratio; CI, Confidence Interval.

**p < 0.01, *p < 0.05.

### Child factors

#### Health and morbidity factors

The proportion of children with the first episode of acute malnutrition who had poor neurodevelopmental outcomes was 31.93% (*n* = 38), while the proportion of those who had the first episode of acute malnutrition but had normal neurodevelopmental outcomes was 34.46% (*n* = 51). The proportion of children who had acute malnutrition relapse after treatment and had poor neurodevelopmental outcomes was 40.33% (*n* = 48). The proportion of children who had acute malnutrition relapse after treatment and had normal neurodevelopmental outcomes was 27.70% (*n* = 41). The proportion of children with low birth weight and poor neurodevelopmental outcomes was 30.25% (*n* = 36). The proportion of children who had low birthweight but had normal neurodevelopmental outcomes was 28.38% (*n* = 42).

There was a significant association between acute malnutrition relapse after treatment and neurodevelopmental outcomes [COR = 1.99; CI:1.09, 3.62; *p*-value: 0.04]. However, there was no association between the first episode of acute malnutrition and neurodevelopmental outcomes [COR = 1.26; CI:0.69, 2.31; *p*-value: 0.54]. Based on the study assumption that children with first episode of acute malnutrition have similar neurodevelopmental outcomes as children with normal neurodevelopmental outcomes, there was a significant association between relapse and neurodevelopmental outcomes [COR = 1.76; CI:1.06, 2.95; *p*-value: 0.03]. Similarly, based on the study assumptions, the association between first episode and neurodevelopmental outcomes was insignificant [COR = 0.89; CI:0.53, 1.49; *p*-value: 0.66]. The association between child maturity and neurodevelopmental outcomes was significant [COR = 3.75; CI:1.60, 8.81; *p*-value:<0.01]. There was no association between birthweight and neurodevelopmental outcomes [COR = 1.09; CI:0.64, 1.86; *p*-value: 0.74], as shown in Table 4.

**Table 4:** Association between child health and mortality factors and neurodevelopmental outcomes.

	Normal neurodevelopment *n* (%)	Poor neurodevelopment *n* (%)	COR	95% CI	*P*-value
First episode					
Normal nutrition status	56 (62.92)	33 (37.08)	1.99	1.09, 3.62	0.04
Relapse	51 (34.46)	38 (31.93)			
Total	89 (100)	89 (100)			
Relapse					
Normal nutrition status	56 (62.92)	33 (37.08)	1.26	0.69, 2.31	0.54
First episode	41	48			
Total	89 (100)	89 (100)			
First episode and non-first episode (Non first episode = Normal nutrition status+ relapse)					
Non-first episode	97 (65.54)	81 (68.07)	0.89	0.53, 1.49	0.66
First episode	51 (34.46)	38 (31.93)			
Total	148 (100)	119 (100)			
Relapse and no relapse (No relapse = normal nutrition status + first episode)					
No relapse	107 (72.30)	71 (59.66)	1.76	1.06, 2.95	0.03*
Relapse	41 (27.70)	48 (40.33)			
Total	148 (100)	119 (100)			
Birthweight					
High birth weight	106 (71.62)	83 (69.75)	1.10	0.64, 1.86	0.73
Low birth weight	42 (28.38)	36 (30.25)			
Total	148 (100)	119 (100)		1.60, 8.81	
Child maturity at birth					
Term	140 (94.59)	98 (82.35)	3.75	1.60, 8.81	<0.01*
Preterm	8 (5.41)	21 (17.65)			
Total	148 (100)	119 (100)			

COR, Crude Odds Ratio; CI, Confidence Interval.

**p < 0.01, *p < 0.05.

#### Nutritional and behavioral factors

The proportion of children who did not reach minimum dietary diversity and had poor neurodevelopmental outcomes was 43.70% (*n* = 52). The proportion of children who did not reach minimum dietary diversity but had normal neurodevelopmental outcomes was 30.41% (*n* = 45). Then proportion of children who were not exclusively breastfed and had children with poor neurodevelopmental outcomes was 45.38% (*n* = 54). The proportion of children who were not exclusively breastfed but had children with normal nutrition status was 25.68% (*n* = 38). There was a significant association between both dietary diversity and exclusive breastfeeding with neurodevelopmental outcomes [COR = 1.78; CI:1.07, 2.94; *p*-value: 0.03], and [COR = 2.40; CI:1.44, 4.03; *p*-value: <0.01], respectively. There was also a significant association between breastfeeding initiation and neurodevelopmental outcomes [COR = 2.16; CI:1.15, 4.04; *p*-value: 0.02], as shown in Table 5.

**Table 5:** Association between child nutrition and behavioral factors and neurodevelopmental outcomes.

	Normal neurodevelopment *n* (%)	Poor neurodevelopment *n* (%)	COR	95% CI	*P*-value
Diet diversity					
Reached minimum dietary diversity	103 (69.59)	67 (56.30)	1.78	1.07, 2.94	0.03*
Didn’t reach minimum dietary diversity	45 (30.41)	52 (43.70)			
Total	148 (100)	119 (100)			
Exclusive breastfeeding					
Exclusive breastfeeding	110 (74.32)	65 (54.62)	2.41	1.44, 4.03	<0.01**
No exclusive breastfeeding	38 (25.68)	54 (45.38)			
Total	148 (100)	119 (100)			
Initiation of breastfeeding					
Early initiation	128 (86.49)	89 (74.79)	2.16	1.15, 4.04	0.02*
Late initiation	20 (13.51)	30 (25.29)			
Total	148 (100)	119 (100)			

COR, Crude Odds Ratio; CI, Confidence Interval.

**p < 0.01, *p < 0.05.

### Maternal factors

#### Maternal health

The proportion of primary caregivers who were malnourished and had children with poor neurodevelopmental outcomes was 9.24% (=11). The proportion of the primary caregivers with normal nutrition status but with children with poor neurodevelopmental outcomes was 90.76% (*n* = 108).

The proportion of primary caregivers who had postpartum depression and had children with poor neurodevelopmental outcomes was 31.93% (*n* = 38). The proportion of the primary caregivers who did not have postpartum depression but had poor neurodevelopmental outcomes was 68.07% (*n* = 81), as shown in Table 6. There was a significant association between both maternal nutrition status and postpartum depression [COR = 2.41; CI:0.86, 6.72; *p*-value: 0.09], and [COR = 3.62; CI:1.92, 6.83; *p*-value: < 0.01], respectively.

**Table 6:** Association between maternal health factors and neurodevelopmental outcomes.

	Normal neurodevelopment *n* (%)	Poor neurodevelopment *n* (%)	COR	95% CI	*P*-value
Maternal nutrition status					
Normal nutrition status	142 (95.95)	108 (90.76)	2.41	0.86, 6.72	0.09*
Malnourished	6 (4.05)	11 (9.24)			
Total	148 (100)	119 (100)			
Postpartum depression					
No postpartum depression	131 (88.51)	81 (68.07)	3.62	1.92, 6.83	<0.01**
Postpartum depression	17 (11.49)	38 (31.93)			
Total	148 (100)	119 (100)			

COR, Crude Odds Ratio; CI, Confidence Interval.

**p < 0.01, *p < 0.05.

### Household factors

#### Economic and financial factors

The proportion of households that were food insecure and had children with poor neurodevelopmental outcomes was 64.71 (*n* = 77). In contrast, the proportion of households that were food secure but had children with poor neurodevelopmental outcomes was 35.29% (*n* = 42). There was a significant association between food security and neurodevelopmental outcomes [COR = 1.65; CI:1.00, 2.70; *p*-value: 0.04] as shown in Table 7.

**Table 7:** Association between household economic and financial factors and neurodevelopmental outcomes.

	Normal neurodevelopment *n* (%)	Poor neurodevelopment *n* (%)	COR	95% CI	*P*-value
Food security					
Food secure	70 (47.30)	42 (35.29)	1.65	1.00, 2.70	0.04*
Food insecure	78 (52.70)	77 (64.71)			
Total	148 (100)	119 (100)			

COR, Crude Odds Ratio; CI, Confidence Interval.

**p < 0.01, *p < 0.05.

#### Logistic regression of factors associated with neurodevelopmental outcomes among children in Nairobi informal settlements

Acute malnutrition relapse after treatment was a significant predictor of neurodevelopment outcomes. Children who had either never relapsed from acute malnutrition after treatment or had normal nutrition status were 2.08 times more likely to have normal neurodevelopmental outcomes compared to children who had relapsed after treatment [AOR = 2.08; CI:1.11, 3.90; *p*-value: 0.02]. The first episode of acute malnutrition was not a predictor of neurodevelopmental outcomes [AOR = 1.06; CI:0.56, 2.01; *p*-value: 0.86]. Other significant predictors of neurodevelopment outcomes among children under 5 years in Nairobi informal settlements were maternal postpartum depression and child maturity at birth (either term or preterm). All these significant predictors (Relapse, maternal postpartum depression and child maturity at birth) were also significant in the bivariate analysis using the COR.

Postpartum depression was a significant predictor of neurodevelopmental outcomes. Children whose mothers did not have postpartum depression were 3.62 times more likely to have normal neurodevelopmental outcomes [AOR = 3.62; CI:1.86, 7.04; *p*-value: <0.01]. Children’s maturity at birth was a significant predictor of neurodevelopmental outcomes. Term babies were 2.93 times more likely to have normal neurodevelopmental outcomes compared to preterm babies [AOR = 2.93; CI:1.21, 7.12; *p*-value: 0.02] as shown in Table 8. The qualitative findings further established that relapse in acute malnutrition after treatment affects children’s neurodevelopmental outcomes. Such children often have delayed developmental milestones, which include poor school performance.

**Table 8:** Logistic regression of factors associated with neurodevelopmental outcomes among children.

Neurodevelopment	AOR	St. Err.	*t*-value	*p*-value	95% CI		Sig
First episode	1.06	0.35	0.17	0.86	0.56	2.01	
Relapse	2.08	0.67	2.30	0.02	1.11	3.90	*
Postpartum depression	3.62	1.23	3.79	0.00	1.86	7.04	**
Child maturity	2.93	1.33	2.37	0.018	1.21	7.12	*
Constant	0.10	0.06	−3.79	0.00	0.03	0.33	**
Mean dependent var		0.55	SD dependent var		0.50		
Pseudo r-squared		0.08	Number of obs		267		
Chi-square		30.45	Prob > chi2		0.00		
Akaike crit. (AIC)		346.54	Bayesian crit. (BIC)		364.47		

AOR, Adjusted Odds Ratio; CI, Confidence Interval.

**p < 0.01, *p < 0.05.

“*He's just okay, but when you compare him with other children, you find that other children of his age are far ahead, but we just have hopes that one day we'll also get there*”. CG10_Healthcare provider

“*For school-going children who are malnourished, when the child reaches the stage of studying, their concentration goes down*”. CG6_Caregiver

It was reported that sanitation and diarrhea, were associated with neurodevelopmental outcomes. The mother’s level of education also determines whether the child will have a normal neurodevelopment or not.

“*The child will be affected because if the environment in which the child is raised has poor sanitation standards, it means the child will not grow due to exposure to more contamination that will lead to vomiting and diarrheal. Such an environment will hinder the child’s development*.” CG10_Caregiver

A healthcare provider also alluded that poor sanitation within the communities was one of the contributors of relapse and poor neurodevelopment.

“*It is evident that there are high rates of relapse and children who have delayed developmental milestones in this community. The underlying issue is the poor sanitation, which can lead to diarrhea further complicating the situation*”. CG07_Healthcare provider

The study participants reported that breastmilk was very important for children’s neurodevelopment.

“*Yes, you find that a mother breastfeeding the child must follow the nutritional guidelines provided on how she is supposed to breastfeed the child. If the mother does not breastfeed the child as recommended, the child usually develops other health effects because breastmilk is renowned for being the best for child growth, child development, and child behavior*.” KII_CHV_2_Community health volunteer

## Discussion

This study established that children who had acute malnutrition relapse after treatment had delayed neurodevelopmental outcomes compared to normal children and those who did not relapse after treatment. These findings were also supported by qualitative findings, which further emphasized the association between relapse and delayed neurodevelopmental outcomes as reported by caregivers and healthcare workers. The first episode of acute malnutrition was not a predictor of poor neurodevelopment. However, earlier studies had shown that an association acute malnutrition in general (both first episode and relapse combined) was associated with poor neurodevelopment outcomes (McCormick et al., 2020). This may be because of the repeated episodes exacerbating nutrient deficiencies, impairing brain plasticity, and prolonging periods of vulnerability during critical stages of brain development. Therefore, programs by the governments and organizations aimed at improving the neurodevelopmental outcomes among children under 5 years should focus on relapsed cases more than children having acute malnutrition for the first time.

In addition to relapse in acute malnutrition after treatment, we found that postpartum depression was a significant predictor of delayed neurodevelopmental outcomes in the under-5 children. These findings are in line with previous studies, which found that children whose mothers had postnatal depression were more than five times likely to have delayed language development, emotional development, and cognitive development (Ali et al., 2013). Data from the qualitative findings also showed the association between maternal postpartum depression and delayed neurodevelopmental outcomes. Therefore, postpartum depression among women should be managed effectively to prevent their children from having poor neurodevelopmental outcomes. The government and relevant stakeholders should therefore integrate nutrition interventions with maternal mental health support. Term babies were more likely to have normal neurodevelopmental outcomes compared to preterm babies. A similar study done earlier also found that neurodevelopmental outcomes improved in term babies compared to preterm babies (Hua et al., 2022). Additionally, it showed that neurodevelopmental outcomes improved with a rise in gestational weeks (Hua et al., 2022).

The qualitative and quantitative findings showed that, there was an association between food insecurity and poor neurodevelopmental outcomes. However, earlier studies are also in line with this the qualitative insights. An earlier study showed that food insecurity had a long-lasting effect on cognitive development and behaviors (Gallegos et al., 2021). This implies that inadequate food security may hinder proper cognitive and physical development in children, potentially being associated with long-term deficits. The qualitative findings further supported these findings as the study participants reported the association between food insecurity and poor neurodevelopmental outcomes. Therefore, prioritizing food security in public health and developmental policies are essential to foster optimal neurodevelopment and improve overall child well-being.

Our qualitative findings showed that there was an association between exclusive breastfeeding and neurodevelopmental outcomes. Breastfeeding provides essential nutrients and antibodies that boost a baby’s immune system, promoting healthy growth and development. It also strengthens the bond between mother and child while offering long-term health benefits for both. This finding is in line with an earlier study that showed that exclusive breastfeeding has a significant positive association with children’s neurodevelopmental outcomes (Onyango et al., 2022).

The qualitative findings further established that there was an association between sanitation and poor neurodevelopmental outcomes. By the time we were conducting the literature search, there was no study that had investigated the association between sanitation and children’s neurodevelopmental outcomes. Our findings imply that strengthening sanitation interventions could play a critical role in improving neurodevelopmental outcomes among acute malnutrition relapses. Therefore, integrating targeted sanitation and hygiene programs into child health and nutrition initiatives is recommended to mitigate the risk of poor neurodevelopment.

The qualitative findings showed that there was an association between diarrhea and poor neurodevelopmental outcome. By the time we were conducting the literature search, there was no study that had investigated the direct link between diarrhea and children’s neurodevelopmental outcomes. These are interesting findings as they imply that preventing and effectively managing diarrheal episodes is essential to safeguarding children’s neurodevelopmental outcomes. The ministry, county governments, and program implementers should aim toward strengthening access to clean water, sanitation, hygiene (WASH) interventions, and timely treatment of diarrheal diseases to reduce the risk of poor neurodevelopmental outcomes among children.

This research addresses a critical knowledge gap in the association between relapse and neurodevelopmental outcomes and focuses on the potential long-term consequences of relapse, which are often under-researched. Children under five who experienced relapses after treatment had delayed neurodevelopmental outcomes compared to those who did not relapse, indicating that relapse cases are at greater risk of long-term developmental deficits. The study had a limitation of dealing with some temporal confounders due to its design. Interventions aimed at improving developmental outcomes among children in informal settlements should prioritize relapse cases to break the cycle of malnutrition and ensure more effective support for optimal cognitive and physical development, ultimately reducing the broader societal and economic impacts of developmental delays.

## Conclusion

Our study demonstrates that children under five who experience relapse after treatment for acute malnutrition exhibit significantly delayed neurodevelopmental outcomes compared to those who do not relapse, highlighting the urgent need by the local and national governments and the non-governmental organizations to prioritize relapse cases in intervention programs. Moreover, factors such as postpartum depression, preterm birth, food insecurity, lower socioeconomic status, suboptimal diet quality, and inadequate breastfeeding practices further compound these developmental delays, reinforcing the importance of integrated maternal and child health strategies. Targeted public health interventions that address these multifaceted determinants are essential to breaking the cycle of malnutrition and promoting optimal neurodevelopment in vulnerable populations.

## Data Availability

The raw data supporting the conclusions of this article will be made available by the authors, without undue reservation.
